# The more ambidexterity the better? The moderating effect of organizational learning between high-performance HR practices and organizational performance

**DOI:** 10.3389/fpsyg.2023.1283637

**Published:** 2024-01-03

**Authors:** Canhao Wang, Meng Zhang, Hongtao Ma

**Affiliations:** ^1^Administration and Management Institute, Ministry of Agriculture and Rural Affairs School, Beijing, China; ^2^Business School, Beijing Normal University, Beijing, China; ^3^Business School, Shandong Normal University, Jinan, China; ^4^The Tourism College of Shanghai Normal University, Shanghai, China

**Keywords:** high-performance HR practices, organizational ambidexterity, organizational learning, organizational performance, SMEs

## Abstract

**Objective:**

The objective of this study is to test the directly impact of high-performance HR practices on organizational performance, and the mediating effect of organizational ambidexterity empirically. Moreover, the moderating role of organizational learning in the relationship between ambidexterity and specialization in exploitation or exploration on firm performance has also been examined. Ultimately, we construct a moderated mediation model.

**Methods:**

Questionnaires were distributed to the target enterprises mainly through the contacts of the research group members, the local management consulting association and the training opportunities for leaders. Finally, a total of 347 CEO questionnaire data were collected from Chinese SMEs. The sample cover Shanghai, Beijing, Chongqing, Jiangsu, Zhejiang, Guangdong, Henan, Sichuan and other eastern and central regions. SPSS 23.0 and AMOS 24.0 were used to analyze the data.

**Results:**

The results revealed that high-performance HR practices had a positive effect on organizational performance and that organizational ambidexterity played a partially mediating role between high-performance HR practices and organizational performance. Further, organizational learning moderated the effects of organizational ambidexterity and organizational specificity on firm performance.

**Discussion:**

This study provided valuable practical insights. On one hand, this study provides a concrete operational scheme for SMEs in China to realize organizational ambidexterity by integrating a series of HR practices such as employees’ ability, motivation and opportunity. On the other hand, through organizational ambidexterity, firms can not only obtain organizational long-term performance by enhancing their new product R & D capabilities, that is, exploratory innovation, but also utilize their existing resources to improve and expand their existing products and services, that is, to achieve short-term performance by exploitative innovation.

## 1 Introduction

At present, the wave of economic globalization has deepened the economic ties between countries, and it has also intensified competition in domestic and foreign markets. For enterprises, in order to hire and retain excellent talents to gain their own competitive advantages, it is inseparable from the efficiency of human resources and human resource management. Therefore, effective management of organizational talent is widely recognized as a crucial factor for organizations to improve their competitiveness.

Over the past two decades, the strategic role of high-performance human resource (HR) practices has received considerable attention in management literature, e.g., the impact of high-performance HR practices on individual (Wood et al., 2012; Ma et al., 2021) and organizational performance (Kroff et al., 2017; Kirkpatrick and Hoque, 2022). Although most previous studies have confirmed the positive relationship between high-performance HR practices and organizational performance from behavioral, human capital, and resource-based perspectives, some scholars have questioned this hypothesis and reached opposite conclusions (Richard and Johnson, 2001; Batt and Colvin, 2011; Gardner et al., 2011). The inconclusive findings suggest that the theoretical logic underlying the mechanisms linking high-performance HR practices and organizational performance remains fragmented, and a deeper understanding of the relationship between the two is needed. This paper contributes to the existing literature in two aspects: First, focus on mediating factors that link high-performance human resource practices to corporate performance, e.g., organizational ambidexterity; second, explore the contextual factors (organizational learning) that may influence the impact of high-performance HR practices on firm performance.

Organizational ambidexterity originated from March (1991) and Tushman and O’Reilly (1996). They extended the exploration versus exploitation construct to define a new typology of technological innovation strategy along two generic dimensions: exploration innovation and exploitative innovation. If an organization scores high in both exploratory and developmental innovation strategies, we can consider it as organizational ambidexterity. In this case, the product of the two scores will be a good proxy measure of organizational ambidexterity (He and Wong, 2004). In this study, we attempt to examine the mediating role of organizational ambidexterity between high-performance HR practices and organizational innovation performance for the following reasons: First of all, More and more research on strategic human resource management (SHRM) has recognized employees at the individual level as important sources of competitive advantage for enterprises and believed that a system of human resource practices may enable firms to develop ambidexterity (Patel et al., 2013; Mom et al., 2019; Gürlek, 2021). For example, Swart et al. (2019) raised the senior employees are more likely to use “integration”, “role expansion” and “tone setting”, whilst employees with specialist knowledge about their clients use “gap filling” to enable ambidexterity. Furthermore, despite the general assumption that exploration and exploitation in organizational ambidexterity are often inconsistent or even contradictory, a series of theories and methods are adopted to solve the conflict between exploration and exploitation, such as different leadership styles at the individual level (Wang and Duan, 2018), top management team behavioral integration at the team level (Lubatkin et al., 2006) and organizational learning at the organizational level (Xu and Li, 2013), few studies have examined the role of human resource practices in the process of realizing organizational ambidexterity. Finally, although previous researches have confirmed that high-performance HR practices is the most direct prerequisite for organizational performance (van Esch et al., 2021), the internal mechanism of how high-performance HR practices affects organizational performance remains to be further explored, this manuscript attempts to examine the mediating role of organizational ambidexterity within this comprehensive framework.

In addition, this study suggests that there may be some situational factors, such as organizational learning, that enhance the positive relationship between organizational ambidexterity and organizational performance. Organizational learning refers to a process of acquiring, absorbing, integrating and applying internal and external knowledge and skills, and regards it as a dynamic capability that affects organizational innovation performance (Baker and Sinkula, 1999; Carmeli et al., 2010). First of all, due to the lack of theoretical support, there are still many uncertainties in the relationship between organizational ambidexterity and organizational performance. For example, there is a positive correlation between organizational ambidexterity and organizational performance (Voss and Voss, 2013), and the inverse U-shaped influence (Caspin-Wagner et al., 2012) and negative correlation (Junni et al., 2013). These results show that there is a contingency effect between organizational ambidexterity and organizational performance. Unfortunately, however, there are few studies on this contingency effect. Furthermore, organizational learning can effectively help enterprises identify new internal and external information, and better manage cognitive overload caused by organizational ambidexterity through knowledge transformation, which enables organizations to not only utilize existing knowledge to improve their profitability, but also develop new knowledge to enhance their ability to adapt to new radical changes, ultimately achieving a synergistic effect between exploration and utilization. Therefore, organizational learning is beneficial for enterprises to more effectively acquire, absorb, transform, and apply knowledge, which is conducive to overcoming the trade-offs between simultaneous exploration and exploitation, promoting the advantages of organizational ambidexterity, and transforming it into an improvement in organizational performance. In conclusion, this paper attempts to examine the moderating role of organizational learning between organizational ambidexterity and organizational performance, which can serve as a trigger for organizations to prefer a certain strategy to a certain extent. Additionally, recent researches on other internal and external contingency factors between organizational ambidexterity and organizational performance mainly includes environmental uncertainty (Uotila et al., 2009), organizational redundant resources (Fu et al., 2016), organizational dynamic environment (Chang, 2016), absorptive capacity (Solís-Molina et al., 2018) and dynamic capabilities (Jin et al., 2019).

**Figure 1:**
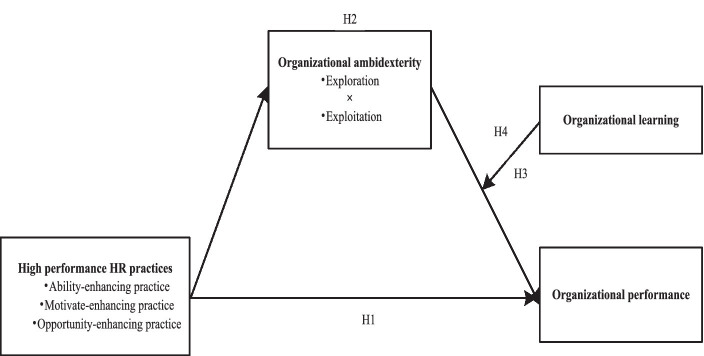
Theoretical research model.

Therefore, drawing on the strategic human resource management theory, strategic management theory (this article conceptualizes organizational ambidexterity as a strategic perspective), and contingency theory (organizational learning is seen as an intrinsic contextual variable), this study reveals the mediating role of organizational ambidexterity between high-performance HR practices and organizational performance, and takes organizational learning as a moderating variable to further investigate the relationship between organizational ambidexterity and organizational performance. Ultimately, we constructs a moderated mediation model as shown in Figure 1.

## 2 Theoretical background and hypothesis development

### 2.1 Strategic human resource management

In recent years, with the increasing market environment of VUCA and market competition, based on the original human resource management theory, strategic human resource management has gradually become a hot topic in academia and industry (Sun et al., 2007). Drawing on the resource-based view, strategic human resource management scholars argue that high-commitment human resource strategy gains a competitive advantage by create a larger pool of enterprise-level human resources that are unique and valuable. Different from traditional human resource management that has been critiqued for its failure, or conceptual inability (Harney and Collings, 2021), strategic human resource management is shifting to a more nuanced conceptualization and measurement of HR practices (e.g., flexibility, job design, etc.), which emphasizes the construction of reasonable human resource practices by influencing employees’ attitudes and behaviors (Boon et al., 2019). Consequently, the research of strategic human resource management examines the impact of high-performance HR practices on organizational performance. Bamberger and Meshoulam (2000) believed that high-performance HR practices were composed of a series of policies and measures that can significantly enhance employees’ working ability, motivation and participation opportunities. Sun et al. (2007) divided the dimensions of high-performance HR practices into three dimensions: ability-enhancing practice, motivation-enhancing practice, and opportunity-enhancing practice, which included eight aspects. The current researches on high-performance HR practices are mainly based on the results-oriented to examine its impact on the individual level and organizational level (Castanheira and Story, 2016; Iyanda Ismail et al., 2021; el-Kassar et al., 2022; Hauff et al., 2022). Therefore, based on the theory of human strategic human resource management, this study attempts to investigate the internal mechanism of high performance human resource management practices on organizational performance.

### 2.2 Organizational ambidexterity

Ambidextrous organizations advocated for having two different abilities, i.e., exploratory ability and exploitative ability, to enhance their core competitiveness in an increasingly competitive environment (Duncan, 1976). March (1991) innovatively divided organizational learning into exploratory learning and exploitative learning. Subsequently, Tushman and O’Reilly (1996) raised organizational ambidexterity to the theoretical level based on March’s research, pointing out that organizations should not only meet the needs of external environmental changes (exploratory ability), but also focus on the ability to meet current organizational needs (exploitative ability), which means that organizational ambidexterity must meet the needs of both current and future development of organizations. Current researches on organizational ambidexterity mainly focuses on antecedents including individual level, team level and organization level (Alghamdi, 2018; Luo et al., 2018; Chen et al., 2019; Mammassis and Kostopoulos, 2019; Guo et al., 2022; Wang et al., 2023). Specifically, at the individual level, the researchers examined the impact of different leadership styles on organizational ambidexterity. For example, at the individual level, CEO, as a key decision maker for the success and development of the organization has played a guiding role in organizational ambidexterity (Wang et al., 2023). At the team level, it mainly examines the impact mechanism of diversity, heterogeneity and cognitive structure of top management team on organizational ambidexterity. For example, Chen et al. (2019) believed the time horizon mean and diversity possessed by TMT can individually and interactively influence organizational ambidexterity. Similarly, at the organizational level, most studies on organizational dualism mainly focus on organizational strategy, organizational structure and organizational context (Jansen et al., 2009; Alghamdi, 2018). Hughes et al. (2021) proposed the term “innovation ambidexterity” and examined the impact of strategic entrepreneurship on innovation ambidexterity and expected that subsequent innovation ambidexterity affected profitability in the young technology-based firms. Generally, organizational structure is considered as the macro antecedent of organizational ambidexterity, while organizational context is the micro antecedent of organizational ambidexterity. Therefore, this study tries to combine organizational ambidexterity with high-performance HR practices to investigate the mechanism of organizational performance.

### 2.3 From high-performance HR practices to organizational ambidexterity: establishing the links

Previous studies have shown different ways to achieve organizational ambidexterity (O’Reilly and Tushman, 2013; Heracleous et al., 2017; Umans et al., 2018). Organizational ambidexterity means that employees can decide when to focus on exploratory activities and when to focus on exploitative activities, which enables employees to pursue both exploratory activities and exploitative activities simultaneously, thus providing theoretical possibilities to promote the realization of organizational ambidexterity from the perspective of high performance HR practices at the employee level.

Currently, the impact of high performance HR practices on organizational ambidexterity mainly lies in the consistency and adaptability necessary to generate organizational ambidexterity. Such as staff recruitment, selection and training as the main form of ability-enhancing HR practices and performance management, incentive compensation as the main form of motivation-enhancing HR practices will motivate employees to achieve organizational expectations, and induce employees to fight for more ambitious goals by establishing the common aspiration and collective identity, which will help employees to create value in the short term, thus promoting the consistency of organizational ambidexterity. Opportunity-enhancing HR practice, mainly in the form of internal promotion opportunities, job security, information sharing and decision-making participation will make employees believe that they are the most valued member of the organization, which will help to cultivate trust among employees and form a good organizational atmosphere; In addition, by providing smooth promotion channels, organizations can make employees willing to take risks to explore and innovate. Therefore, opportunity-enhancing HR practice promotes the adaptability of organizational ambidexterity. To some up, ability-enhancing HR practice and motivation-enhancing HR practice can help employees acquire the knowledge, skills and abilities to effectively fulfill current job demands in a productive manner (exploitive activities); Opportunity-enhancing HR practice can bring a greater sense of trust and support within the organization. In this situation, it is conducive to knowledge sharing and exchange among employees, thus helping the organization to put forward more innovative solutions (exploratory activities). A recent study on the Spanish hotel industry has confirmed that a series of activities such as ability, motivation and opportunity contained in high-performance HR practices will positively affect organizational ambidexterity, for example, ability-enhancing HR practice can not only closely match personal skills with job requirements to pursue short-term value creation, but also help employees participate in decision-making and information sharing to pursue exploratory innovation, that is, ability-enhancing HR practice can promote organizational exploitative activities and exploratory activities simultaneously; Based on the data of 84 multinational M&A enterprises from emerging economies, Rao-Nicholson et al. (2020) confirmed that high performance HR practices have a positive impact on organizational ambidexterity. Based on the above, we propose that high-performance HR practices may promote the realization of organizational ambidexterity. Therefore, we hypothesize the following:

*H1:* High-performance HR practices is positively correlated with organizational ambidexterity.

### 2.4 The mediating role of organizational ambidexterity

In this research, we refer to the research of Bamberger and Meshoulam (2000), and divide high-performance human resource practices into three dimensions: ability-enhancing practice, motivation-enhancing practice and opportunity-enhancing practice. First, ability-enhancing HR practice including staffing and employee training will directly affect employees’ work efficiency through their knowledge, skills and work ability. Through systematic training, the recruited employees can transform their existing knowledge and skills into new knowledge, and constantly expand the breadth and depth of knowledge to create new organizational knowledge pool, which is also crucial to the improvement of organizational performance (van Esch et al., 2021). Second, motivation-enhancing HR practices generally include formal performance appraisal, skill-based compensation and incentive compensation, these competitive compensation initiatives can attract and retain more valuable talent. Moreover, skill-based compensation and incentive compensation will provide incentives for the extra effort and are often positively related to employees’ organizational citizenship behavior (van Esch et al., 2021). Therefore, employees’ awareness of self-learning and self-skill improvement will be further enhanced, and they will more inclined to apply their skills to organizational performance (Chiang and Birtch, 2012). Finally, opportunity-enhancing practice is found that when employees are given autonomy to participate in the strategic decision-making of the organization, they may be more willing to take risks, and try to actively seek new organizational solutions to handle the challenges, which will effectively meet the needs of organizational performance improvement. Therefore, this paper believes that high-performance HR practices may promote the realization of organizational ambidexterity. For example, Zhou et al. (2021) confirmed the positive correlation between organizational ambidexterity and organizational performance.

High performance HR practices are a set of management measures and means aimed at influencing employees’ attitudes and behaviors, thereby further affecting organizational performance. However, generally speaking, the improvement of organizational performance does not directly come from high-HR practices themselves (Crook et al., 2011; Ployhart and Moliterno, 2021), but from the application of these human resource practices and the ability of employees to pursue both exploratory and exploitative activities, that is, organizational ambidexterity. Specifically, both the cultivation of exploitative capability and exploratory capability depend on the strategic goal of the enterprise as the guidance and direction. Moreover, the matching organizational resources are also required to provide guarantee and support for the realization of organizational ambidexterity. In particular, high-performance HR practices can bring process advantages such as learning and innovation to organizations, thus promoting the improvement of organizational performance. Finally, according to the resource-based view, high-performance HR practices can also improve organizational performance by developing and testing key internal capabilities, such as organizational ambidexterity, so that organizations can obtain lasting competitive advantages (Chadwick and Cappelli, 1999). For example, on one hand, high-performance HR practices can improve organizational short-term performance by improving the knowledge, skills and ability of employees to maintain and consolidate the existing market and reduce the operating cost of the enterprise. On the other hand, high-performance HR practices can also improve employees’ risk-taking and exploratory abilities by providing them with internal promotion opportunities, job security, information sharing, and decision-making participation, which will help organizations to have more innovative solutions to enhance their flexibility and defense against market changes, thus contributing to the sustainable dynamic competitiveness.

Based on the above, this study constructs a complete chain of high-performance HR practices-organizational ambidexterity-organizational performance. In other words, the positive effects of high-performance HR practices may flourish in the form of both exploration and exploitation capabilities or outcomes, i.e., organizational ambidexterity. Therefore, we hypothesize the following:

*H2:* The positive effect of high-performance HR practices on firm performance is mediated by organizational ambidexterity.

### 2.5 The moderating role of organizational learning

Organizational ambidexterity, as an organization’s ability to pursue exploratory innovation and exploitative innovation at the same time, has long been considered important for the survival and success of organizations (Kafetzopoulos, 2021; Solís-Molina et al., 2022). However, there is also some empirical evidence that organizational ambidexterity has mixed effects on performance, i.e., there are complex relationships of positive correlation, negative correlation and even no correlation between organizational ambidexterity and organizational performance, these findings are consistent with the concern of Gupta et al. (2006), that is, organizational ambidexterity is sometimes ineffective for organizational performance, which also makes organizational specificity strategy focusing on either exploratory activities or exploitative activities better than organizational ambidexterity. When organizations adopt a specific strategy, they can concentrate on a large amount of resources in a certain field, thus avoiding the tensions that arise from competing for an organization’s limited resources. However, organizational specificity also has its shortcomings, which may lead organizations to fall into the “success trap” caused by excessive attention to exploitative activities, or may also lead organizations to fall into the “failure trap” due to excessive attention to exploratory activities, resulting in the negative effect of self-reinforcing brought by exploratory activities and exploitative activities. So, under what circumstances, which strategy (organizational ambidexterity and organizational specificity) will have a more positive impact on organizational performance? Therefore, this paper attempts to investigate the contingency effect of organizational learning between organizational ambidexterity and organizational performance.

Organizational learning theory holds that enterprises can help themselves identify the value of new internal and external information through organizational learning and apply it to business purposes, which enables organizations to improve organizational performance through interaction with the environment and other companies at the inter-organizational level. The current measurement of organizational learning is mainly based on Baker and Sinkula’s (1999) research, which divides organizational learning into three dimensions: vision sharing, open mind, and learning commitment. Considering that this research examines the moderating effect of organizational learning, in order to avoid unnecessary confusion, we treat organizational learning as a single structure without separately hypothesizing and testing the effects of each dimension. Based on the knowledge transformation path related to organizational learning ability proposed by Fernhaber and Patel (2012), this study argues that when the organizational learning level is high, organizations can better manage the cognitive load caused by organizational ambidexterity, so that organizations can not only utilize the existing knowledge to improve their profitability in the technology cycle, but also develop new knowledge to enhance company’s ability to adapt to new radical changes, and ultimately achieve synergy between exploration and exploitation. Therefore, high-level organizational learning can enable enterprises to acquire, absorb, transform and apply knowledge more effectively, enable companies to overcome the trade-offs between exploration and exploitation simultaneously, promote the advantages of organizational ambidexterity, and finally translate it into the improvement of organizational performance.

On the contrary, at low organizational learning level, enterprises are unable to effectively absorb and utilize internal and external knowledge simultaneously, they have to only concentrate limited resources on exploration or exploitation. Specifically, at the initial stage of the enterprise life cycle, due to the fact that the new technology has not yet developed to a mature commercial application stage, combined with the low level of organization learning, the knowledge required for exploratory activities has not been fully developed. In this case, if precious resources are invested in exploitative activities, a lot of time and money will be wasted. Therefore, at low organizational learning level, organizational specificity strategy that specialize in exploratory activities are more effective than organizational ambidexterity. However, in the mature period of technology, the core technology mastered by enterprises has matured and there is no room for further development. In this case, if enterprises blindly pursue exploration, they will fall into a vicious circle of failure. Therefore, it will be more beneficial for the organizational performance to focus on exploitive activities. In addition, the low level of organizational learning means that organizations cannot acquire existing knowledge and new knowledge at the same time, that is, enterprises are unable to integrate exploration and exploitation. Ultimately, at low organizational learning level, organizations pursuing exploration and exploitation simultaneously will reduce organizational performance. Ebben and Johnson (2015) proved that when an enterprise pursues a specific strategy, it will achieve better performance than pursuing two or more strategies at the same time. Therefore, in enterprises with low organizational learning level, organizational specificity strategy is preferable to organizational ambidexterity.

In conclusion, this paper argues that, at low organizational learning level, organizational specificity, which focuses on exploratory activities or exploitative activities, can improve organizational performance more than organizational ambidexterity. On the contrary, at high-level organizational learning, organizations can not only improve the existing knowledge pool, but also absorb and digest new knowledge and implement knowledge innovation, thus helping to realize the synergy between exploratory activities and exploitative activities. Organizational ambidexterity can achieve better organizational performance than organizational specificity strategy.

Therefore, we hypothesize the following:

*H3:* Organizational learning positively moderates the positive relationship between organizational ambidexterity and organizational performance.

In order to better compare and analyze the contingency effect of organizational learning in the process of organizational ambidexterity and organizational specificity on organizational performance, we further propose the following hypothesis based on H3:

*H3a:* At a high-levels of organizational learning, organizational ambidexterity is more effective in improving organizational performance than organizational specificity strategy that only focuses on exploitative activities.

*H3b:* At high-levels of organizational learning, organizational ambidexterity is more effective in improving organizational performance than organizational specificity strategy that only focuses on exploratory activities.

*H3c:* At low-levels of organizational learning, organizational specificity strategy focusing only on exploitative activities is more effective in improving organizational performance than organizational ambidexterity.

*H3d:* At low-levels of organizational learning, organizational specificity strategy focusing only on exploratory activities is more effective in improving organizational performance than organizational ambidexterity.

Based on the above hypothesis, when the level of organizational learning is high, the high-performance HR practices such as capability enhancement practice, motivation enhancement practice and opportunity enhancement practice can be better applied to the consistency and adaptability of organizational ambidexterity. For example, when the level of organizational learning is high, employees often tend to possess rich knowledge, skills, and abilities to effectively meet their current work needs in a productive manner (exploitative activities). At the same time, based on the good organizational learning atmosphere formed within the enterprise, it is conducive to knowledge sharing and communication among employees, thereby helping the organization propose more innovative solutions (exploratory activities) to ultimately promote the improvement of organizational performance. Therefore, we believe that with the improvement of organizational learning level, high-performance human resource practices can better enhance organizational performance through organizational ambidexterity. Finally, we propose hypothesis 4:

*H4:* Organizational learning positively moderates the mediating effect of organizational ambidexterity on the relationship between high-performance HR practices and organization performance.

A moderated mediation framework and hypotheses are illustrated in Figure 1.

## 3 Methodology

### 3.1 Sample and data collection

To test the hypotheses, questionnaire survey was used in this paper. Drawing on the existing scales in foreign literature, we adopted the method of two-way translation to construct the initial scale. In order to better fit the management problems in the Chinese context, this paper first conducted a pre-test. Based on 78 valid questionnaires collected from the predictive test, we analyzed the reliability and validity of the initial scale and further modified the original scale according to the results. In addition, we also invited four scholars with rich theoretical background and senior managers with front-line practical experience to put forward their own opinions and suggestions on the questionnaire items, and the formal scale was constructed finally. The formal investigation was conducted from September 2020 to February 2021. In view of existing studies, compared with large enterprises, it is more instructive to explore how to achieve organizational ambidexterity and improve organizational performance of SMEs. In this study, the senior managers of SMEs were selected as the research object. The questionnaire targets were obtained through the social relationship of research members, enterprise management consulting associations and training opportunities for leaders. The distribution methods were adopted by hand and online. A total of 611 target enterprise CEO questionnaire data were collected. The incomplete and invalid questionnaires were eliminated, finally, a valid questionnaire was obtained from 347 SMEs, with a questionnaire efficiency of 56.8%. The samples cover Shanghai, Beijing, Chongqing, Jiangsu, Zhejiang, Guangdong, Henan, Sichuan and other eastern and central regions, and the potential impact of geographical differences is mitigated to some extent.

### 3.2 Measurement

The used measurements in this paper is based on the foreign mature scale, and forms the final questionnaire through the pre-test method. Except for control variables, all variable items are anchored on a 5-point Likert scale, ranging from “1” to “5,” representing “strongly disagree” to “strongly agree,” respectively.

#### 3.2.1 High-performance HR practices

At present, the measurement of high performance HR practices is mainly derived from three comprehensive measures:(1) turnover, including staffing, career security, selection and training; (2) evaluation and rewards, including flexible work design, performance appraisal, incentive compensation and internal promotion; and (3) employment relations, including job design and employee participation. We used the scale developed by Sun et al. (2007) to measure high-performance HR practices with a total of 22 items from three aspects of employees’ ability, motivation and opportunity.

#### 3.2.2 Organizational ambidexterity

Currently, the most widely used measure of organizational ambidexterity is the five-point Likert-type scale by He and Wong (2004). The scale is mainly composed of eight items. The first four items measure the company’s exploratory activities, and the last four items are related to the company’s exploitative activities. Based on an accurate calculation of organizational ambidexterity must account for both balance and magnitude but must also correct the flaw in the balance calculation, we draw on the measurement method of Hughes et al. (2021), and the final mathematical calculation for innovation ambidexterity is

$$\mathrm{Innovation Ambidexterity}=\sum \left(\left(\mathrm{Explore}\times \mathrm{Exploit}\right)-\sqrt{{\left(\mathrm{Explore}-\mathrm{Exploit}\right)}^{2}}\right)\text{.}$$

#### 3.2.3 Organizational learning

About the measurement of organizational learning, there are multiple maturity questionnaire. We adopted the scale developed by Baker and Sinkula (1999), which had been proved to have strong reliability and validity by a large number of studies. It divided organizational learning into three dimensions, including vision sharing, open mind and learning commitment. Vision sharing consisted of four items, learning commitment and open mind had three items respectively, 10 items in total. It is worth noting that we treat organizational learning as a single structure and do not discuss the moderating effects of the three dimensions separately.

#### 3.2.4 Organizational performance

Considering that the research object of this paper is SMEs, and most of them are non-listed enterprises, it is difficult to obtain indicators of organizational performance from the public database, such as the number of corporate patents and R&D costs. Therefore, based on the organizational performance scale revised by Jiménez-jiménez and Sanz-valle (2008), we adopted subjective measurement method to investigate the overall operation of enterprises in the past 3 years in terms of market share, profitability, productivity level and customer satisfaction, with a total of four items.

#### 3.2.5 Control variables

Considering that the nature of the enterprise, age of establishment, size of firm and industry type may have an impact on organizational ambidexterity and organizational performance, this paper takes them as control variables. We divide the nature of enterprises into two categories: private enterprises and non private enterprises. The size of firm is reflected in the natural logarithm of the number of each firm. Firm age is reflected in the natural logarithm of the number of years it has been established +1. We controlled for industry type onto profitability and respondents self-identified their industry. Descriptive statistics and correlations of variables are shown in Table 1.

**Table 1:** Descriptive statistics and correlation matrix.

Variable	Mean	SD	1	2	3	4	5	6	7	8
Corporate nature	2.22	0.96	1							
Firm age	5.62	1.98	0.084	1						
Firm size	4.44	1.62	0.103	0.431^**^	1					
Industry type	4.04	2.37	−0.055	0.028	−0.055	1				
HPHRP	3.74	11.68	−0.041	−0.109^*^	−0.012	−0.082	**0.709**			
OA	3.91	5.38	0.002	−0.054	−0.032	−0.018	0.404^*^	**0.795**		
OL	3.54	6.70	−0.002	−0.099	−0.017	−0.061	0.515^**^	0.527^**^	**0.757**	
OP	3.81	3.05	0.040	0.180*	0.076	−0.050	0.569^**^	0.496^**^	0.553^**^	**0.836**

The diagonal elements (in bold) are the square root AVE of each variable.*p < 0.05, **p < 0.01.HPHRP, High-performance HR practices; OA, Organizational ambidexterity; OL, Organizational learning; OP, Organizational performance.

## 4 Results

### 4.1 Reliability and validity

Exploratory factor analysis (EFA) was employed to assess the reliability of the scales. We calculated Cronbach’s α and composite reliability (CR) scores. First, based on the reliability analysis, the Cronbach’s α values of high-performance HR practices, organizational ambidexterity, organizational learning and organizational performance were 0.929, 0.862, 0.904 and 0.819, respectively, and the value of each variable exceeded the threshold level of 0.70, providing adequate internal consistency.

Second, we also calculated the KMO values and the Bartlett values of each variable to confirm whether scales are suitable for factor analysis. The KMO of high-performance HR practices, organizational ambidexterity, organizational learning and organizational performance were 0.930, 0.902, 0.917, and 0.787, respectively, which met the threshold of 0.7, and the Bartlett values had a statistically significant level (Sig = 0.000), which indicated that our study was suitable for factor analysis. Subsequently, we used principal component analysis and the maximum variance rotation method to calculate the factor loadings (See the Appendix). All items had statistically significant loadings of over 0.50, indicating high convergent validity. In terms of explaining the total variation, the variance contribution rate of each variable was more than 50%, indicating that the scales had good construct validity.

Finally, based on the factor loadings and we calculated CR and showed that the values for all the variables ranged from 0.903 to 0.956, which exceeded the threshold level of 0.60, once again proving that the scales had good reliability. In addition, the AVE square root of each variable calculated is greater than the correlation coefficient of the row and column, as shown in the diagonal of Table 1. Every value exceeded the 0.50 cutoff, indicating that the study had high convergent validity.

In terms of discriminant validity, confirmatory factor analysis was used to test. First, the four latent variables, i.e., high-performance HR practices, organizational ambidexterity, organizational learning and organizational performance, involved in this paper are taken as the reference model. Then, through the induction and integration of the above four latent variables, four competition models including three-factor model^a^, three-factor model^b^, two-factor model and single-factor model are finally generated, respectively. Finally, *γ*^2^/df, RMSEA, NFI, CFI, and TLI were used to illustrate the fitting indices of the established models. As shown in Table 2, in the four-factor model, all the fitting indicators accepted the requirements (RMSEA < 0.08, NFI > 0.90, CFI > 0.90, TLI > 0.90) and were statistically significant compared with other nested models (three-factor model^a^, three-factor model^b^, two-factor model, and single-factor model), which further indicated that this study had significant discriminant validity.

**Table 2:** Confirmatory factor analysis results.

**Models**	*γ* ^2^	df	γ^2^/df	RMSEA	NFI	CFI	TLI
Five-factor model	338.693	128	2.646	0.069	0.905	0.938	0.928
Four-factor model	342.523	129	2.646	0.066	0.902	0.925	0.928
Three-factor model^a^	544.104	132	4.122	0.085	0.852	0.884	0.864
Three-factor model^b^	368.786	132	2.794	0.076	0.901	0.923	0.918
Two-factor model	749.481	134	5.493	0.105	0.803	0.821	0.792
Single-factor model	843.616	135	6.249	0.121	0.768	0.793	0.762

Five-factor model: high-performance HR practices, organizational ambidexterity, organizational learning, organizational performance, non-measurable methodological factor; Four-factor model: high-performance HR practices, organizational ambidexterity, organizational learning, organizational performance; Three-factor model^a^: high-performance HR practices + organizational ambidexterity, organizational learning, organizational performance; Three-factor model^b^: high-performance HR practices + organizational learning, organizational ambidexterity; organizational performance; Two-factor model: high-performance HR practices + organizational ambidexterity, organizational ambidexterity + organizational performance; Single-factor model: high-performance HR practices + organizational ambidexterity + organizational learning + organizational performance. “+” means fusion.

### 4.2 Common method variance

This study adopted the unmeasurable latent method factor technique to address common method variance (CMV) concerns. First, based on the four factor model, the common method variance factor (CMV) is entered into the structural equation model as a potential variable to construct a five factor model, and its variance is set as “1,” and the load path of CMV affecting each index variable is set as “a.” Next, by testing the fitting indicators of the five-factor model, we could verify whether there is a common method variance. The judgment criteria are as follows: after adding the unmeasurable latent method factor, if the fitting indices are significantly improved, such as when the values of CFI, TLI and NFI increase by more than 0.1, and the values of RMSEA and RMR decrease by more than 0.05, it indicates that there is a serious common method variance. As shown in Table 2, compared with the reference model, i.e., the four-factor model, the fitting indices of the five-factor model do not improve, and the results do not meet the above judgment criteria, indicating that the fitting indices are not significantly improved after adding the unmeasurable latent method factor. Therefore, this study believes that there is no serious common method variance.

### 4.3 Tests of hypotheses

We used tolerance and variance inflation factor (VIF) to test multi-collinearity problems. The results showed that the tolerance of each variable was great than 0.1, and the VIF values were less than 2, which were far below the threshold level of 10. Therefore, there is no serious multi-collinearity problem in this study. Then, hierarchical regression analysis was used to estimate the above hypotheses.

#### 4.3.1 The main effect test

Based on the model M3, we introduced four control variables and the independent variable of high-performance HR practices into the regression equation as shown in Table 3. According to the model M4, the high-performance HR practices had a significantly positive impact on organizational performance (*β* = 0.598, *p* < 0.001). Therefore, *H1* is supported.

**Table 3:** Regression analysis of high-performance practices and organizational performance.

Variable	Organizational ambidexterity		Organizational performance		
	M1	M2	M3	M4	M5
**Control variables**					
Corporate nature	0.007	0.035	0.022	0.046	0.041
Firm age	−0.068	0.017	0.183^***^	0.255^***^	0.253^***^
Firm size	−0.013	−0.041	−0.008	−0.032	−0.026
Industry type	−0.021	0.034	−0.054	−0.007	−0.012
**Independent variables**					
High-performance HR practices		0.698^***^		0.598^***^	0.481^***^
**Mediating variables**					
Organizational ambidexterity					0.121^***^
*F*	0.524	65.207	3.152	42.780	36.228
*R* ^2^	0.006	0.421	0.036	0.385	0.401
△*F*	0.524	322.11	3.125	182.047	6.552
△*R*^2^	0.006	0.415	0.036	0.339	0.016

*p < 0.05, **p < 0.01, ***p < 0.001.

#### 4.3.2 The mediating effect test

Referring to the research of Baron and Kenny (1986), the test steps of this paper were as follows: First, the main effect of high-performance HR practices and organizational performance was estimated; then, we examined the direct effect of high-performance HR practices on mediating variable, i.e., organizational ambidexterity. Last, we incorporated high-performance HR practices and organizational ambidexterity into the regression equation simultaneously to test the mediating effect.

As shown in Table 3, the model M2 showed that high-performance HR practices had a significant positive effect on organizational ambidexterity (*β* = 0.698, *p* < 0.001). Moreover, we found that the model M4 supported the positive impact of high-performance HR practices on organizational performance (*β* = 0.598, *p* < 0.001). The model M5 incorporated high-performance HR practices and organizational ambidexterity into the regression equation simultaneously. Regression result showed that organizational ambidexterity had a significant positive effect on organizational performance (*β* = 0.121, *p* < 0.001). Further, the influence of high-performance HR practices on organizational performance was still significantly positive (*β* = 0.481, *p* < 0.001), which meant that organizational ambidexterity played a partial mediating role between high-performance HR practices and organizational performance. Therefore, *H2* was also supported.

#### 4.3.3 The moderating effect test

By introducing two-way interactions of organizational learning with exploitation and exploration, and a three-way interaction with organizational ambidexterity, this paper attempted to compare the different effects of organizational ambidexterity and organizational specificity on organizational performance and test the moderating role of organizational learning. Following Voss and Voss (2013), the impact of organizational ambidexterity on organizational performance was estimated as the effect of increasing exploitation (or exploration) when exploration (or exploitation) are set at high levels. Likewise, the impact of specialization in exploitation (or exploration) was estimated as the effect of increasing exploration (or exploitation) with exploration (or exploitation) set at low levels. This allowed us to compare the effect on organizational performance of organizational ambidexterity with the effect of specialization in exploitation or exploration.

This paper used hierarchical regression method to estimate three nested models. To avoid multi-collinearity due to the presence in the same equation of first order and interaction terms, the VIFs after centralized were all below the critical value of 10. Ultimately, three nested model with hierarchical regression analysis were constructed, as shown in Table 4. First, the model M6 incorporated the control variables such as corporate nature, firm age, firm size and industry type into the regression equation to test its impact on organizational performance. Then, the model M7 introduced variables such as exploratory activities, exploitative activities and organizational ambidexterity on the basis of model M6. Subsequently, the model M8 included organizational learning, interactive items of organizational learning and exploratory activities, organizational learning and exploitative activities, organizational learning and organizational ambidexterity, respectively. According to model M7, the regression coefficient of the interaction between organizational learning and exploratory activities on organizational performance was positive (*β* = 0.048, *p* < 0.05), indicating that organizational learning positively moderated the impact of exploratory activities on organizational performance; On the contrary, the regression coefficient of the interaction between organizational learning and exploitative activities on organizational performance was negative (*β* = −0.136, *p* < 0.05), indicating that organizational learning negatively moderated the impact of exploitative activities on organizational performance; Finally, the regression coefficient of the interaction between organizational learning and organizational ambidexterity on organizational performance was positive (*β* = 0.228, *p* < 0.01), indicating that organizational learning positively moderated the impact of organizational ambidexterity on organizational performance. Therefore, *H3* and *H4* are preliminarily supported.

**Table 4:** Results of regression analysis of the moderating effect of organizational learning.

Variable	Organizational performance			
	M6	M7	M8	VIF
**Control variables**				
Corporate nature	0.022	0.022	0.012	1.028
Firm age	0.183^**^	0.218^***^	0.203^***^	1.290
Firm size	−0.008	−0.030	−0.021	1.336
Industry type	−0.054	−0.039	−0.016	1.018
**Independent variables**				
Exploratory activities		0.367^***^	0.204^**^	2.655
Exploitative activities		0.254^***^	0.171^*^	2.595
Organizational ambidexterity		0.146^**^	0.049	5.985
**Moderating variable**				
Organizational learning			0.479^***^	1.789
**Interaction**				
Organizational learning*Exploration			0.048^*^	4.015
Organizational learning*Exploitation			−0.136^*^	5.386
Organizational learning*Ambidexterity			0.228^**^	7.224
*F*	3.170	19.723	20.045	
*R* ^2^	0.036	0.286	0.418	
△*F*	3.170	39.487	18.673	
△*R*^2^	0.036	0.250	0.132	

*p < 0.05, **p < 0.01, ***p < 0.001.

#### 4.3.4 The moderated mediating effect test

To test organizational learning in moderating the mediating effect of organizational ambidexterity between high-performance HR practices and organizational performance, we continued to use bootstrapping procedures (with 5,000 samples), and the confidence interval was also 95%. Based on the mean value of moderating variables ± SD, we distinguished two categories from organizational learning: high organizational learning and low organizational learning.

The results of moderated by organizational learning. As shown in Table 5, under the organizational learning level of ± S.D., the indirect impact of high-performance HR practices on organizational performance through organizational ambidexterity is 0.004 when organizational learning is at a high level, and the 95% bootstrap confidence interval excludes 0 (LLCI = 0.0264, ULCI = 0.2388). The results of this analysis confirm that at a high level of organizational learning, high-performance HR practices through organizational ambidexterity has a significant positive indirect effect on organizational performance. In addition, when organizational learning is at a low level, the indirect impact of high-performance HR practices on organizational performance through organizational ambidexterity is 0.002. The 95% bootstrap confidence interval includes 0 (LLCI = −0.0166, ULCI = 0.0219), which indicates that at a low level of organizational learning, the indirect effect of high-performance HR practices on organizational performance through organizational ambidexterity is not significant. Moreover, at different levels of organizational learning, the mediating effect of organizational ambidexterity is significantly different (∆γ = 0.10, *p* < 0.01). Together, organizational learning positively moderates the mediating role of organizational ambidexterity between high-performance HR practices and organizational performance. Therefore, *H4* is supported.

**Table 5:** Analysis of the moderated mediation model.

Moderating variable	Moderated by organizational learning			
	Direct effect	Indirect effect	Direct effect 95% CI	Indirect effect 95% CI
**Mediation condition**				
High organizational learning	0.112	0.004	[−0.0119, 0.1567]	[0.0264, 0.2388]
Low organizational learning	−0.030	0.002	[−0.0380, 0.0905]	[−0.0116, 0.0219]

Source: collated by authors.

### 4.4 Supplemental analyses

**Figure 2:**
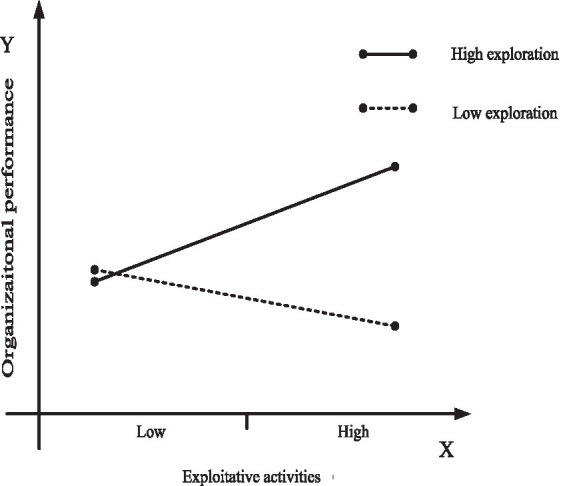
Plotting of the effect of exploitative activities on organizational performance under high-levels of organizational learning.

**Figure 3:**
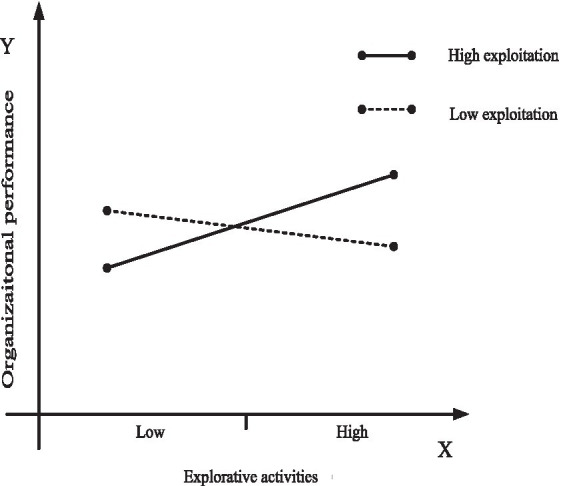
Plotting of the effect of explorative activities on organizational performance under high-levels of organizational learning.

**Figure 4:**
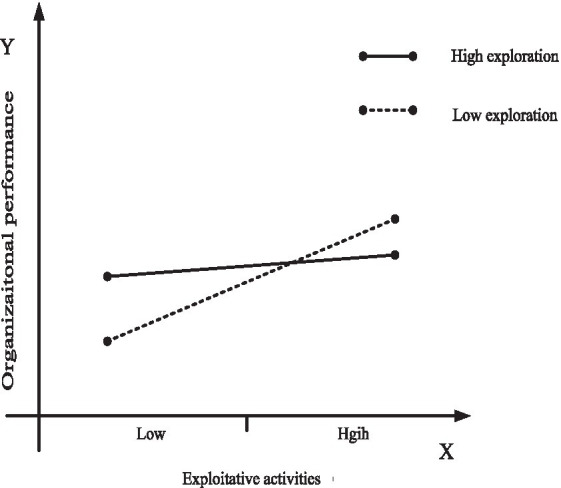
Plotting of the effect of exploitative activities on organizational performance under low-levels of organizational learning.

**Figure 5:**
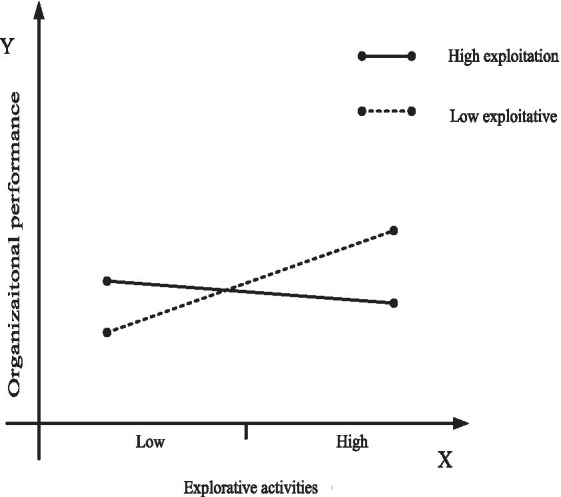
Plotting of the effect of explorative activities on organizational performance under low-levels of organizational learning.

To further investigate the moderating role of organizational learning, we use marginal analysis to estimate how increasing exploitation in settings involving exploration, and increasing exploration in settings involving exploitation, affect organizational performance. To arrive at a clearer presentation of the results, we use Figures 2, 3 to plot the moderating effect of organizational ambidexterity and organizational specificity on organizational performance obtained at high levels of organizational learning. Figures 4, 5 do the same at low levels of organizational learning. In each figure, the continuous line plots the effect on organizational performance of increasing organizational ambidexterity by increasing exploitation (or exploration) while exploration (or exploitation) is set at high levels; On the other hand, the dashed line represents the effect on organizational performance of increasing organizational specificity by increasing exploitation (or exploration) when exploration (or exploitation) is set at low levels. Each figure presents the comparison between the effects on organizational performance of organizational ambidexterity and organizational specificity.

#### 4.4.1 The influence of organizational ambidexterity and specialization in exploitation or exploration (organizational specificity) on organizational performance when organizational learning level is high

Hypothesis *H3a* and *H3b* propose that at high-levels of organizational learning, organizational ambidexterity has a greater impact on enterprise performance than organizational specificity focusing on exploratory activities or exploitative activities. Marginal analysis results show that the effect on organizational performance of increasing exploitation activities at high levels of exploration (i.e., achieve organizational ambidexterity by increasing exploitative activities) is positive and significant, as shown by the continuous line in Figure 2. On the other hand, the effect on organizational performance of organizational specificity (specialization in exploitative activities) is assessed by calculating the effect of increasing exploitative activities at low levels of exploration. The value is negative significantly (dashed line in Figure 2). As can be seen in Figure 2, firms with high levels of organizational learning benefit more from pursuing both exploratory activities and exploitative activities (organizational ambidexterity) than from a specialization in exploitative activities (organizational specificity). Hence, *H3a* is further supported.

Likewise, still at high-levels of organizational learning, the effect on organizational performance of increasing explorative activities at high levels of exploitation (i.e., achieve organizational ambidexterity by increasing explorative activities) is positive and significant, as shown by the continuous line in Figure 3. Moreover, specialization in explorative activities (organizational specificity), consisting on increasing explorative activities at low levels of exploitation has a negative role on organizational performance (dashed line in Figure 3). This reflects that organizational ambidexterity is more effective than specialization in explorative activities (organizational specificity) at high levels of organizational learning. Therefore, *H3b* is further supported.

Overall, at high levels of organizational learning, the impact of organizational ambidexterity on organizational performance is higher than that of organizational specificity focusing on explorative activities or exploitative activities. This shows that organizational learning plays an important role in solving the trade-offs between exploitation and exploration. Organizations can achieve higher performance by pursuing exploratory activities and exploitative activities simultaneously.

#### 4.4.2 The influence of organizational ambidexterity and specialization in exploitation or exploration (organizational specificity) on organizational performance when organizational learning level is low

Hypothesis *H3c* and *H3d* hold that at low-levels of organizational learning, specialization in exploitative activities or explorative activities (organizational specificity) is more effective on organizational performance than organizational ambidexterity. Again, using marginal analysis, the results show that increasing exploitation activities at high levels of exploration (achieve organizational ambidexterity by increasing exploitative activities) has a significant positive impact on organizational performance (continuous line in Figure 4), while increasing exploitative activities at low levels of exploration (achieve organizational specificity by increasing exploitative activities) has also a significant positive impact on organizational performance (dashed line in Figure 4). However, it should be noted that although increasing exploitative activities improves organizational performance at different levels of exploration, the slope of dashed line in Figure 4 is much higher than that of the continuous line. Therefore, organizations cannot obtain better organizational performance by adding exploitative activities to their already high exploration efforts when facing low-levels of organizational learning. In this case, the effect of specialization in exploitative activities (organizational specificity) on organizational performance is higher than that of organizational ambidexterity, which supports *H3c*.

Likewise, still at low-levels of organizational learning, increasing explorative activities at high-levels of exploitation (achieve organizational ambidexterity by increasing explorative activities) has a negative effect on organizational performance (continuous line in Figure 5). Instead, the effect of specialization in explorative activities on organizational performance at low levels of exploitation (achieve organizational specificity by increasing explorative activities) is positive and significant, as shown by the dashed line in Figure 5. This confirms that at low levels of organizational learning, organizational specificity (specialization in explorative activities) is more effective than organizational ambidexterity in improving organizational performance. Therefore, *H3d* is also supported.

Finally, this paper concludes that organizational learning moderates the comparative effect of organizational ambidexterity and organizational specificity on organizational performance. Specifically, at high-levels of organizational learning, the impact of organizational ambidexterity on organizational performance is higher than that of organizational specificity focusing on explorative activities or exploitative activities. On the contrary, at low-levels of organizational learning, organizational specificity focusing on explorative activities or exploitative activities has a higher impact on organizational performance than organizational ambidexterity.

## 5 Discussion

Based on the theory of human resource management and organizational ambidexterity, this study examines the mediating role of organizational ambidexterity between high-performance HR practices and organizational performance. Different from the traditional human resource theory that only emphasizes how to enhance employees’ work motivation to improve organizational performance, such as providing employees with incentive compensation and training, etc., this paper believes that high-performance HR practices that integrates employees’ ability, motivation and opportunity can enhance organizational performance by effectively exploiting existing organizational knowledge and exploring new knowledge, that is, organizational ambidexterity. Moreover, we introduce organizational learning as a contingency element to investigate the moderating effect between organizational ambidexterity and organizational performance, and further verify the boundary conditions of organizational ambidexterity, which helps to explain some inconclusive results about the impact of organizational ambidexterity on organizational performance in existing studies.

### 5.1 Theoretical contributions

The findings of this study have three theoretical contributions. First, compared to current research on the effectiveness of human resource management, which mainly focuses on individual and team levels (Martell and Carroll, 1995; Collins and Clark, 2003), there is a lack of systematic research on the impact on organizational levels. Therefore, this article drew on strategic human resource management theory and focused on the impact of high-performance human resource practices on organizational ambidexterity, we also added a research flow based on human resource theory (Ahammad et al., 2019), and ultimately providing a new theoretical perspective for the antecedents of organizational ambidexterity. In addition, our results extended the influence boundary of high-performance human resource practices on organizational ambidexterity at the organizational level and filled the gap in the previous research (Glaister et al., 2015; Kim et al., 2023). Finally, we demonstrated the importance of understanding the status of the HR department within the organization and how closely the HR function is thought to embody the organization.

Second, the revelation of the mechanism of high-performance HR practices on organizational performance enriches the theoretical research of strategic human resource management on organizational performance. Faced with the dilemma of resource shortage, how SMEs can simultaneously pursue exploration and exploitation to achieve organizational ambidexterity, and its process and effectiveness are worth exploring (Jiang et al., 2022). Based on the theory of organizational ambidexterity, this study proposes that SMEs can achieve internal and external innovation to promote organizational performance improvement by simultaneously focusing on the characteristics of exploration and exploitation in the process of implementing human resource strategy. Therefore, this study reveals the “black box” mechanism of high performance HR practices on organizational performance, thus solving the problem of “how to apply” human resource practices in SMEs, and providing a theoretical basis for how to solve the dilemma of resource shortage.

Third, March (1991) is the first scholar to apply organizational learning to the field of organizational ambidexterity. Since then, research on organizational ambidexterity from the perspective of organizational learning has achieved fruitful results (Raisch and Birkinshaw, 2008; Brix, 2019; Arantes and Soares, 2021). However, most of these studies have directly regarded organizational learning as an antecedent of organizational ambidexterity and there are few studies on organizational learning as a moderating variable in organizational ambidexterity. This study creatively compares and analyzes the impact of organizational ambidexterity and organizational specificity on organizational performance, empirically tests the moderating effect of organizational learning, and tries to investigate the contingency effect between organizational ambidexterity and organizational performance. The results show that compared with organizational specificity, organizational ambidexterity can improve organizational performance more effectively at high-levels of organizational learning, while it is opposite in the context of low-level organizational learning. Finally, we construct a moderated mediation model between high-performance HR practices and performance, which promotes the development of the existing researches. Therefore, this study provides theoretical support for investigating the contingency effect of organizational ambidexterity to some extent.

### 5.2 Practical implications

For management practitioners interested in high-performance human resource management and organizational ambidexterity, this study also provides some meaningful practical insights and how these two structures can improve organizational performance. Firstly, the results clearly demonstrate that organizational ambidexterity plays a mediating role between high-performance HR practices and organizational performance. On one hand, the management mechanism that only focuses on developing a single aspect of enterprises in the traditional industrial period can no longer meet the requirements of current organizational innovation. This study provides a concrete operational scheme for SMEs in China to realize organizational ambidexterity by integrating a series of HR practices such as employees’ ability, motivation and opportunity. On the other hand, through organizational ambidexterity, organizations can not only obtain organizational long-term performance by enhancing their new product R&D capabilities, that is, exploratory innovation, but also utilize their existing resources to improve and expand their existing products and services, that is, to achieve short-term performance by exploitative innovation. Ultimately, this study constructs a complete chain of high-performance HR practices, organizational ambidexterity, and organizational performance, which provides a specific solution to the contradictory problem of how to effectively coordinate short-term performance and long-term performance for SMEs in China.

Second, as our results found, a high-level of organizational learning ability will be more conducive to enhancing the positive effect of organizational ambidexterity on organizational performance. Therefore, organizations should direct their effort to building a positive learning atmosphere in the future, so that organizations and employees can timely acquire, absorb, integrate and utilize new knowledge and skills, and continuously improve the organizational learning level.

Finally, this study provides practical support for small and medium-sized enterprises (SMEs) in China on how to improve their performance and enhance their core competitiveness. Specifically, compared with large enterprises, such as state-owned enterprises in China, SMEs are obviously weak in talent training mode, organizational management level and operation stability. Especially in the current world business pattern is in the VUCA era, improving the anti-risk ability and strain capacity of SMEs is the key for Chinese enterprises to realize the transformation and upgrading. High-performance HR practices, as the initial point of enterprise performance improvement, means that enterprises should achieve organizational ambidexterity to enhance organizational short-term and long-term performance simultaneously, which will not only conducive to reduce enterprise management costs, but also help enterprises to obtain new market share. Therefore, this study has important practical implication on how SMEs can effectively improve their performance in the environment of uncertainty, complexity and dynamics within the system framework of organizational ambidexterity.

### 5.3 Limitations and future avenues of research

Although this research offers several theoretical and managerial implications, it also inevitably has some limitations and provides avenues for future studies. First, we choose the term “high-performance HR practices,” which is considered to be the most commonly used. However, considering its various and rich conceptual meaning, previous studies have more other choices and discussions on high-performance HR practices in theory, such as high-performance work system, flexible work system High involvement human resource practices and best human resource practices, etc. Although the connotations represented by these terms are similar, it does not mean that each term is also similar in the internal impact mechanism of organizational ambidexterity. Therefore, in addition to high-performance HR practices, we can further explore the impact mechanism of other HR practices on organizational ambidexterity and organizational performance in the future.

Second, organizational ambidexterity is an extremely complex realization process. With the increasing market competition, only relying on a single level of factors to explain and verify the realization mechanism of organizational ambidexterity is far from satisfying the development requirements of enterprises in the future. Although this research examines the impact on organizational ambidexterity and organizational performance from high-performance HR practices at the organizational level, there is no further discussion on how to conduct cross-level research from multiple theoretical levels. Therefore, future researches can further expand the antecedents of organizational ambidexterity, such as building a cross-level implementation mechanism of organizational ambidexterity, and conducting empirical data verification to continuously enrich the theoretical and practical significance of organizational ambidexterity.

Third, this research operationalized organizational ambidexterity into the product of exploitative scores and explorative scores as a good proxy measure of organizational ambidexterity. However, this measurement cannot truly reflect the degree of imbalance between exploration and exploitation. It is inconsistent with the concept of organizational ambidexterity, which seeks a balance between the two. Therefore, in future research, we can adopt other methods to evaluate organizational ambidexterity, such as the addition or difference of exploratory scores and exploitative scores. Moreover, we suggest that these measurement methods can also be used as robustness checks in future studies. Moreover, given the cross-sectional data collected in this paper, they may not fully represent dynamic causal conclusions. Therefore, longitudinal or time series data can be used in the future to investigate organizational ambidexterity.

## Data availability statement

The raw data supporting the conclusions of this article will be made available by the authors, without undue reservation.

## Ethics statement

The studies involving humans were approved by the Business School, Beijing Normal University. The studies were conducted in accordance with the local legislation and institutional requirements. Written informed consent for participation was not required from the participants or the participants' legal guardians/next of kin in accordance with the national legislation and institutional requirements.

## Author contributions

CW: Investigation, Methodology, Software, Writing – original draft, Writing – review & editing. MZ: Methodology, Writing – review & editing. HM: Data curation, Investigation, Supervision, Writing – review & editing.
